# Comparison of non-transport disc and conventional distraction osteogenesis for mandibular segmental defect reconstruction

**DOI:** 10.1186/s13018-026-06948-4

**Published:** 2026-05-11

**Authors:** Haiyun Lin, Xiaoxia Zhong, Huijuan Shen, Nuo Zhou, Xuanping Huang

**Affiliations:** 1 Department of Oral and Maxillofacial Surgery, College of Stomatology, Guangxi Medical University, Nanning, 530021 People’s Republic of China; 2 Guangxi Key Laboratory of Oral and Maxillofacial Rehabilitation and Reconstruction; Guangxi Key Laboratory of Oral and Maxillofacial Surgery Disease Treatment, Guangxi Clinical Research Center for Craniofacial Deformity, Guangxi Health Commission Key Laboratory of Prevention and Treatment for Oral Infectious Diseases, College of Stomatology, Guangxi Medical University, Nanning, 530021 People’s Republic of China; 3 Department of Prosthodontics, College and Hospital of Stomatology, Guangxi Medical University, Nanning, 530021 People’s Republic of China; 4 Department of Stomatology, The First Affiliated Hospital of Guangxi Medical University, Nanning, 530021 Guangxi People’s Republic of China

**Keywords:** Non-transport disc distraction osteogenesis, Conventional distraction osteogenesis, Osteogenic quality, Segmental mandibular defects

## Abstract

**Background:**

An animal model of non-transport disc distraction osteogenesis (NTDDO) was developed for repairing segmental mandibular defects in dogs. This study aimed to determine whether any difference exists in osteogenesis within the distraction zone between non-transport disc distraction osteogenesis (NTDDO) and conventional distraction osteogenesis (CDO), in order to provide a reference for its clinical application in the repair of segmental mandibular defects.

**Methods:**

Twenty-four dogs were divided into two groups, and animal models of NTDDO and CDO were established respectively.Samples were taken at 0, 2, 4,and 8 weeks of consolidation. The new bone in the distraction zone was observed or detected by gross condition, X-ray film, Micro CT, biomechanical test. The histological manifestation of new bone was observed by HE staining. The expression of the osteoblast marker Runx2, and the endothelial cell marker CD31 were detected by immunohistochemistry and microvessel density was calculated.

**Results:**

The results from gross specimens, radiographs, and HE staining indicated no significant differences in new bone formation within the distraction gap between the NTDDO and CDO groups at any time point. Micro-CT analysis revealed no statistically significant differences in BMD, BV/TV, Tb.Th, Tb.N, or Tb.Sp (*P* > 0.05). Similarly, biomechanical testing showed no significant difference in maximum load (*P* > 0.05). Immunohistochemical staining demonstrated comparable expression levels of CD31 and Runx2, with no significant difference in neovascular density (*P* > 0.05).

**Conclusions:**

Non-transport disc distraction osteogenesis achieves osteogenic quality equivalent to conventional distraction osteogenesis, with inferior alveolar neurovascular resection posing no adverse effects on bone regeneration. The underlying mechanisms, particularly the potential interplay between nerve regeneration and periosteal contributions, warrant further exploration.

## Background

Mandibular segmental defects, typically arising from tumor resection, trauma, infection, or congenital anomalies, disrupt mandibular continuity and result in functional deficits and aesthetic compromise [1]. Restoring continuity is generally considered to require simultaneous reconstruction, which is critical for achieving both anatomical and functional outcomes [2].Distraction osteogenesis (DO), as an endogenous tissue engineering technique, offers distinct advantages by regenerating native bone to bridge continuity defects, thereby circumventing donor site morbidity, graft failure risks, and suboptimal functional recovery associated with autograft procedures [3].

In current clinical applications of DO for mandibular reconstruction, the transport disc technique predominates but presents specific limitations. Potential complications include vascular compromise of the transport segment, resorption at its leading edge, inadequate union with the native bone stump, and displacement of associated dentition during gradual advancement [4–6]. Furthermore, the technical complexity of creating viable transport segments with preserved periosteal attachments – often requiring osteotomies at one or both defect margins – combined with the frequent necessity for patient-specific distractors, imposes practical constraints on its widespread clinical adoption.

Exploring less invasive alternatives with lower morbidity is clinically worthwhile for reconstructing defects limited to the mandibular body or chin, such as those resulting from keratocystic odontogenic tumors or ameloblastoma. Our team recently implemented an innovative approach in a clinical case involving a recurrent odontogenic keratocyst after multiple curettages [7]. Departing from traditional protocols, we eliminated the transport disc design while preserving periosteal integrity. The surgical procedure consisted of segmental resection of the mandible at the site of the keratocyst, followed by direct approximation of the residual bone segments across the defect, and then distraction osteogenesis performed according to a distraction protocol. The patient ultimately achieved successful mandibular reconstruction with good function and contour. For clarity in description, this paper designates the distraction approach without a transport disc as Non-Transport Disc Distraction Osteogenesis (NTDDO). Additionally, the monofocal distraction osteogenesis applied in the treatment of mandibular hypoplasia is termed Conventional Distraction Osteogenesis (CDO).

Distraction osteogenesis requires maximal preservation of periosteal integrity and bone vascularity during osteotomy, which is a widely acknowledged prerequisite for its success [8, 9]. Furthermore, the impairment of DO outcomes by periosteal stripping highlights the indispensable role of the periosteum in bone regeneration [10]. Furthermore, the nervous system plays a significant regulatory role in bone metabolism during osteogenesis and fracture healing [11, 12]. Experimental evidence demonstrates that 1 cm resection of the sciatic nerve in rabbit tibial DO models significantly impairs osteogenesis, highlighting the necessity of intact innervation for effective bone regeneration [13]. Similarly, inferior alveolar nerve resection has been associated with diminished osteogenic quality in the distraction zone [14, 15].

In non-transport disc distraction osteogenesis (NTDDO), mandibular resection involving the inferior alveolar neurovascular bundle is performed while preserving the periosteum, resulting in a distraction zone enriched with periosteal and soft tissue components. Conversely, conventional distraction osteogenesis (CDO) prioritizes protection of the inferior alveolar neurovascular bundle, requiring neoformation of periosteal and soft tissues during the distraction phase. These distinct tissue microenvironments raise unanswered questions regarding potential differences in osteogenic efficacy between the two techniques. This study was designed to test the hypothesis that, under conditions of inferior alveolar neurovascular bundle sacrifice with intact periosteal preservation, NTDDO can achieve bone regeneration quality comparable to that of CDO.

## Methods

### Establishment of mandibular NTDDO and CDO models

#### Ethical approval

All experimental procedures were conducted in accordance with the guidelines of the Animal Experimental Ethics Committee of Guangxi Medical University (Approval No.: 202209270). Twenty-four healthy adult beagles (male, 1-year-old; 15–20 kg) were provided by the Animal Experimental Center of Guangxi Medical University (Nanning, China).

#### Surgical protocol for model establishment

**Fig. 1:**
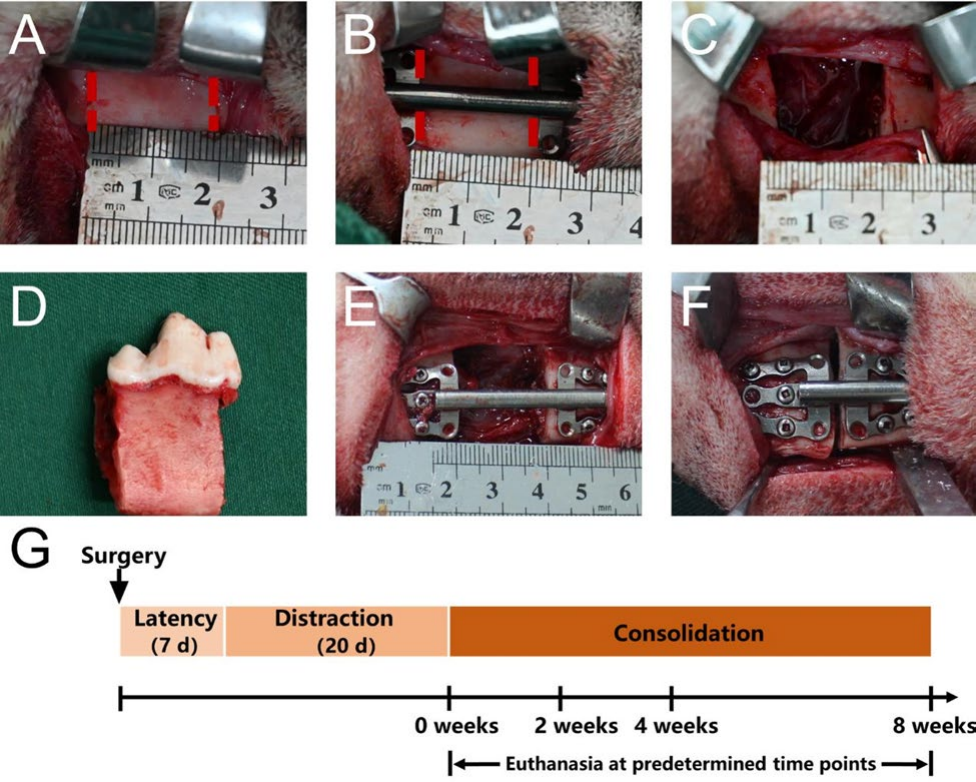
The surgical procedure of the NTDDO model (**A**–**F**) and the distraction protocol (**G**). **A** Osteotomy lines were marked. **B** Preplace the distractor. **C** Mandibular segment resection. **D** Resected mandibular segment with the first molar. **E** Final distractor fixation. **F** Bone stumps brought into contact by the distractor. **G** Distraction protocol

The experimental dogs were randomly divided into two groups, with 12 animals per group. A right mandibular non-transport disc distraction osteogenesis (NTDDO) model was surgically developed in each animal. General anesthesia was administered using a muscarinic antagonist (0.1 mg/kg) and pentobarbital sodium (20 mg/kg), with preoperative local infiltration of lidocaine for analgesia. The buccal surface of the right mandible was surgically exposed. Preoperative markings were made to guide osteotomy and distractor placement. A custom-designed distractor was pre-positioned, followed by a 20 mm segmental osteotomy encompassing the first molar and the inferior alveolar neurovascular bundle without nerve ligation. The distractor was secured with titanium screws (Cibei, China), and the osteotomized bone segments were reapproximated to ensure initial contact (Fig. 1A-F). In contrast to the NTDDO model, the conventional distraction osteogenesis (CDO) animal model was established as follows: After surgically exposing the mandibular bone surface via a buccal approach, a linear osteotomy was performed between the second premolar and first molar without segmental bone resection. The inferior alveolar neurovascular bundle was preserved. Finally, a distraction device was installed.

#### Distraction protocol

The latency period prior to distraction initiation was set at 7 days, consistent with established protocols [16]. Active distraction commenced at a rate of 1.0 mm/day for 20 days, with all time points calculated relative to the completion of the surgical procedure (Fig. 1G).

#### Specimen collection and processing

 At designated consolidation phases (0, 2, 4, and 8 weeks) based on the experimental groups, animals were humanely euthanized with an intravenous overdose of pentobarbital sodium (100 mg/kg) to induce rapid unconsciousness and cardiorespiratory arrest, following the ARRIVE guidelines. Tissue harvesting was subsequently performed for radiographic, histological, and biomechanical evaluations.

### Micro-computed tomography analysis

 The distraction regenerate was scanned using a Latheta LCT-200 micro-CT scanner (Hitachi Aloka Medical, Japan; 80 mm FOV, 40 μm resolution) to acquire X-ray and 3D datasets. BMD was analyzed via integrated software, while morphometric parameters (BV/TV, Tb.Th, Tb.N, Tb.Sp) were quantitatively analyzed using VGStudio Max 2.2 software (Volume Graphics GmbH, Germany).

#### Histological and immunohistochemical staining

 Newly formed bone tissue samples harvested from canine specimens were fixed in 4% paraformaldehyde for 48 h, followed by decalcification in 10% EDTA (pH 7.4) for 2–6 months (duration adjusted according to specimen dimensions). Decalcified tissue blocks were sectioned along the distraction axis using a motorized microtome (Leica RM2235) at 4 μm thickness. Sections underwent hematoxylin-eosin staining (Solarbio, China) for histological analysis.

 For immunohistochemistry, sections were deparaffinized, rehydrated and subjected to high-pressure antigen retrieval in citrate buffer (0.01 mol/L, pH 6.0). Endogenous peroxidase activity was blocked with 3% H_2_O_2_ for 15 min at room temperature, followed by incubation with normal serum. Primary antibodies against Runx2 (1:200; bs-1134R, Bioss, China) and CD31 (1:200; bs-0195R, Bioss, China) were applied overnight at 4 °C. After PBS washing, sections were incubated with horseradish peroxidase conjugated secondary antibody (PV-6000; ZSGB-Bio, China) for 20 min at room temperature (RT), followed by 3,3′-diaminobenzidine (DAB; ZSGB-Bio, China) chromogenic development. Sections were counterstained with hematoxylin, dehydrated, and mounted with resin medium for microscopic analysis.

#### Biomechanical testing protocol

 Mandibular specimens were tested in a three-point bending setup using an Instron E10000 system (Instron, UK). Samples were placed on supports (20 mm span) with central loading aligned longitudinally. A displacement rate of 3 mm/min was applied until failure. Peak bending force (N) was recorded.

#### Microvessel density (MVD)

 Angiogenesis was quantified in CD31-stained sections. Three high-density fields/specimen were identified under low magnification (40×). At 200×, MVD was defined as the mean count of CD31 + endothelial clusters or discontinuous branches across three fields, regardless of lumen presence.

#### Runx2 quantitative analysis

 Digital images were captured at 200× magnification. Three random fields per sample were collected under identical conditions. Quantitative analysis was performed using ImageJ software (NIH, Bethesda, MD, USA). The average optical density (AOD) was calculated as the ratio of integrated optical density to positive area after background subtraction. The mean AOD of three fields per sample was used for statistical comparison between groups (*n* = 3 per group).

### Statistical analysis

 Data were analyzed using GraphPad Prism 10.0 and expressed as mean ± SD. Normality was assessed via Shapiro-Wilk test. Normally distributed data underwent Student’s t-test; non-normal data were analyzed by Mann-Whitney U test. Significance was set at *P* < 0.05 (two-tailed).

## Results

### Gross observations

**Fig. 2:**
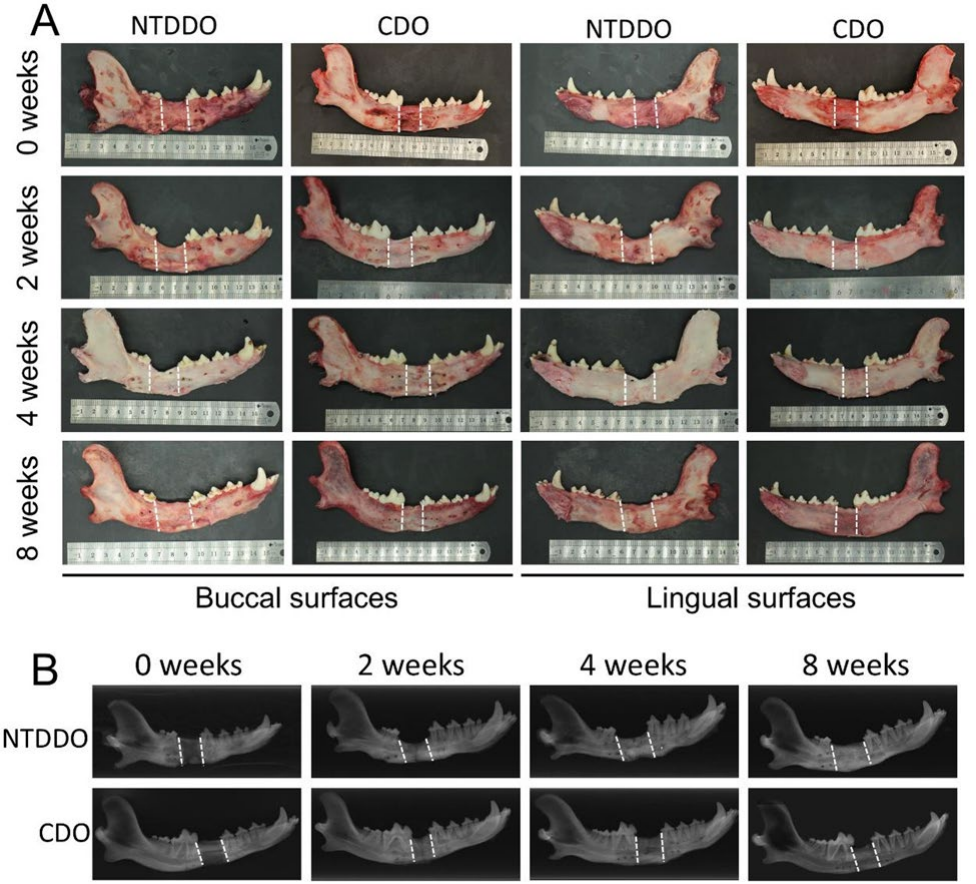
Gross observations and radiographic analysis of NTDDO and CDO groups. **A** Gross specimens demonstrate comparable sequential bone regeneration with similar morphological progression throughout the 0-8-week consolidation period. **B** Radiographic analysis reveals progressive mineralization transitioning from radiolucent osteogenic fronts (0 weeks) to complete osseous integration (8 weeks) with homogeneous bone density, demonstrating closely matched profiles between groups across all timepoints (0, 2, 4, 8 weeks)

 Both NTDDO and CDO groups demonstrated sequential bone regeneration within the distraction gap (Fig. 2A). At 0-week consolidation, the gap was filled with dark-red friable tissue exhibiting clear demarcation from native bone ends, penetrable by surgical scalpel and mechanically unstable. By 2 weeks, partial tissue maturation reduced interface visibility and mobility, though residual instability persisted. Progressive mineralization at 4 weeks achieved mechanical stability with obscured tissue-bone boundaries. Complete bony union resembling native mandibular architecture was observed at 8 weeks; however, NTDDO and CDO groups showed comparable gross morphology across all phases. Lingual osteogenesis demonstrated superior outcomes compared to buccal osteogenesis in the distraction zone across all stages.

### X-ray analysis

 Radiographs demonstrated mineralization progression in the distraction gap, transitioning from radiolucent to radiopaque regions (Fig. 2B). At distraction completion (0-week fixation), radiolucent osteogenic shadows with clear margins were observed at bone stumps. Peripheral osteogenesis with central radiolucent bands persisted at 2 weeks, retaining discernible boundaries. By 4 weeks, central radiolucency diminished with denser mineralization and blurred margins. Full osseous integration matching native bone density occurred at 8 weeks. The radiographic profiles of both groups demonstrated parallel changes over time, from initial assessment (0 weeks) to the final timepoint (8 weeks).

### Micro-CT analysis

**Fig. 3:**
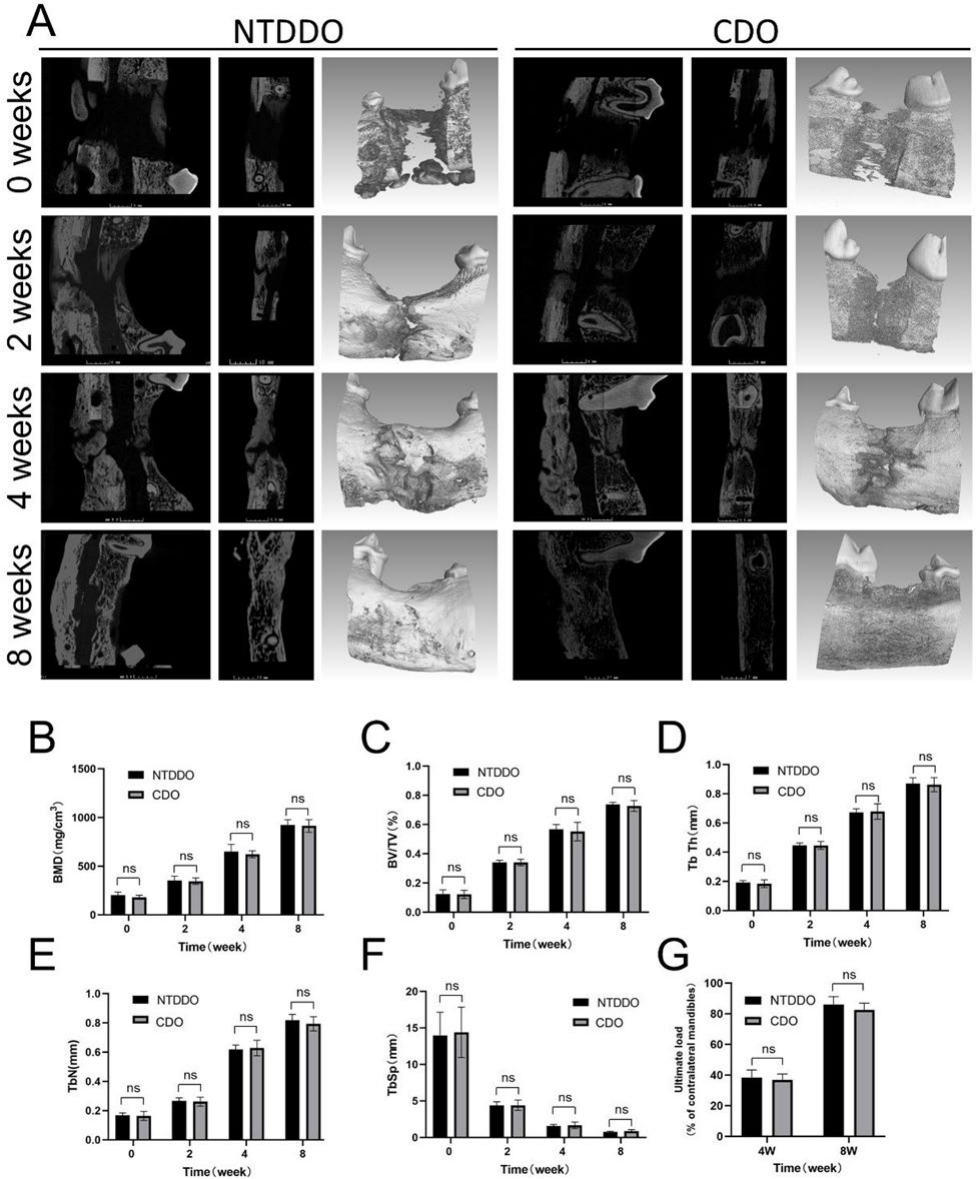
Micro-CT analysis and biomechanical testing of NTDDO and CDO groups. **A** Micro-CT revealed progressive mineralization within the distraction gap, with sparse callus formation at 0-week consolidation transitioning to dense ossification by 8-week. Micro-CT revealed continuity of the inferior alveolar nerve canal across the distraction gap in the NTDDO group, despite transection of the inferior alveolar nerve. **B**–**F** Radiographic analysis reveals progressive mineralization transitioning from radiolucent osteogenic fronts (0 weeks) to complete osseous integration (8 weeks) with homogeneous bone density, showing no significant intergroup differences across all timepoints (0, 2, 4, 8 weeks). **G** biomechanical testing at 4 and 8 weeks revealed marginally higher maximum load (normalized to contralateral bone, %) in NTDDO versus CDO, though statistically insignificant (*P* > 0.05). ns indicates not significant (*P* > 0.05)

 Micro-CT revealed progressive mineralization within the distraction gap, with sparse callus formation at 0-week consolidation transitioning to dense ossification by 8-week (Fig. 3A). Micro-CT revealed continuity of the inferior alveolar nerve canal across the distraction gap in the NTDDO group, despite transection of the inferior alveolar nerve. Both groups exhibited time-dependent increases in bone mineral density (BMD) and morphometric parameters (BV/TV, Tb.Th, Tb.N), accompanied by a reduction in Tb.Sp. No significant intergroup differences were observed in BMD or trabecular indices across all phases (*P* > 0.05; Fig. 3B-F).

### Biomechanical testing

 Mandibular specimens from the distraction zone underwent three-point bending tests at 4- and 8-week consolidation. Maximum load values were normalized to contralateral intact mandibles (expressed as percentage of intact bone strength). While the NTDDO group exhibited marginally higher load-bearing capacity than the CDO group, intergroup differences remained statistically insignificant (*P* > 0.05; Fig. 3G).

### Histological analysis (HE and Masson staining)

**Fig. 4:**
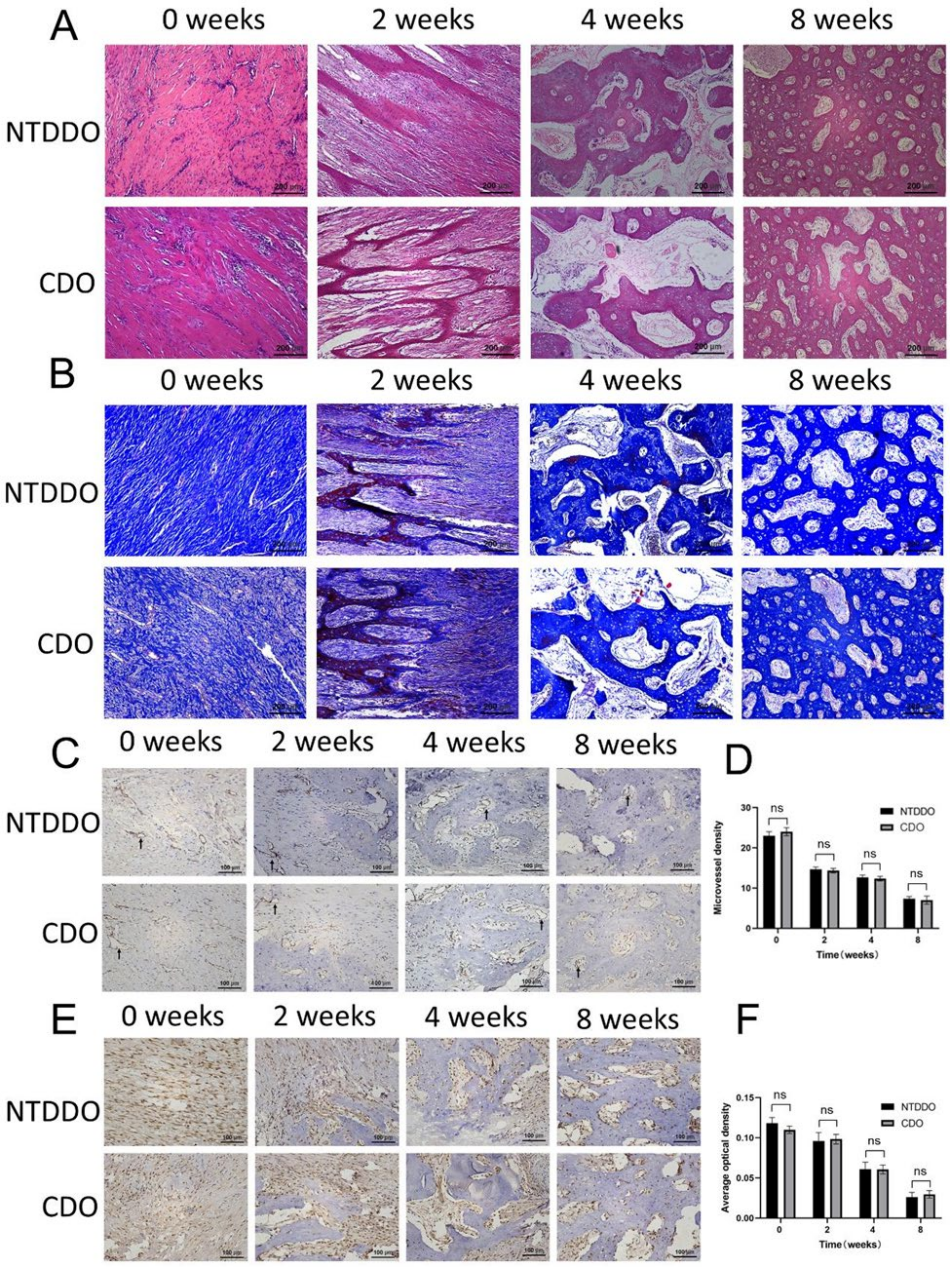
Histological and immunohistochemical Analysis of NTDDO and CDO groups. **A**–**B** The histomorphological progression was remarkably consistent between the two groups, showing no significant disparity in outcomes (**A**: HE staining; **B**: Masson staining). **C**–**D** CD31 immunohistochemical staining results (200×, scale bar, 100 μm, the black arrows represent new blood vessels) revealed a progressive decrease in microvessel density (MVD) correlating with extended consolidation period. Intergroup MVD comparisons showed no statistical significance across phases (*P* > 0.05). **E**–**F** Runx2 immunohistochemical staining, quantified by average optical density (AOD), showed comparable expression levels between the NTDDO and CDO groups at all stages. ns indicates not significant (*P* > 0.05)

Both groups exhibited parallel histomorphological progression (Fig. 4A-B). At 0-week consolidation, the distraction gap was predominantly filled with fibrovascular tissue containing dense collagen bundles and abundant vasculature. By 2 weeks, osteoblast-rich trabeculae emerged within residual fibrous stroma, indicative of active mineralization onset. Transitioning to 4 weeks, mineralized trabeculae became interconnected with complete fibrous tissue resorption. At 8 weeks, mature lamellar bone with well-organized trabecular networks was established. HE and Masson staining showed parallel characteristics in collagen organization, osteoblast density, and mineralization patterns between groups throughout all observed phases (Fig. 4A-B).

### Immunohistochemical analysis

#### CD31 angiogenesis

 CD31 immunostaining revealed intense endothelial cell reactivity within the distraction gap (Fig. 4C). At 0-week consolidation, abundant microvessels permeated the fibrovascular stroma reflecting peak angiogenesis activity. Progressive bone formation was inversely correlated with vascular density, though persistent CD31-positive luminal structures were observed within mineralized trabecular networks. Intergroup MVD comparisons showed no statistical significance across phases. (*P* > 0.05; Fig. 4D).

#### Runx2 osteogenic activity

Runx2 exhibited nuclear localization in osteoblasts and extracellular matrix deposition (Fig. 4E). At 0 weeks, diffuse positivity was observed in fibroblast-like cells and immature osteoid. By week 2, intense nuclear staining concentrated at osteogenic fronts of nascent trabeculae. From 4 to 8 weeks, Runx2 + cells localized specifically to trabecular margins. Runx2 immunohistochemical staining revealed similar intensity between groups throughout all stages (Fig. 4E-F).

## Discussion

 Distraction osteogenesis is a tightly regulated process involving multiple growth factors and cellular interactions [17]. During the latency period, localized trauma-induced inflammatory responses trigger the release of growth factors (e.g., interleukins). These factors subsequently recruit mesenchymal stem cells from bone marrow, periosteum, and endosteum. These cells proliferate and differentiate into osteoblasts and other specialized mesenchymal cells [18]. The osteotomy gap and surrounding hematoma become infiltrated by fibroblasts, chondroblasts, and osteoblasts. These cellular components actively produce extracellular matrix, collagen fibers, and additional growth factors, ultimately forming healing tissue composed of cells, collagen fibers, osteoid, and matrix. Growth factors expressed during the latency period continue to function, reaching peak concentrations during the distraction phase. During the consolidation period, the elevated levels of anabolic growth factors in the regenerate gradually diminish over time [17, 18].

 CD31 immunohistochemical staining (IHC) serves as a pivotal tool for precise identification and localization of endothelial cells, enabling quantitative assessment of microvascular density (MVD), and has become a cornerstone technique for vascular endothelial cell labeling and MVD evaluation. Li et al. [19]demonstrated a robust angiogenic response following distraction osteogenesis (DO), with abundant nascent capillary precursor cells observed within the fibrous bands of the distraction gap. Carvalho et al. [20]further reported extensive neovascularization during bone regeneration in DO. In a canine mandibular DO model, Liao et al. [21] identified a significant surge in newly formed microvessels during the early consolidation phase, followed by a gradual decline in vascular density concomitant with increased trabecular bone formation. Rowe et al. [22] corroborated these findings, revealing that mandibular DO is associated with an intense vascular reaction during the initial distraction phase, whereas vascularity markedly diminishes in the regenerate at later stages.

 Consistent with these reports, our study utilizing CD31 IHC and MVD quantification revealed pronounced angiogenesis during the early fixation phase, with vascular density progressively decreasing over time. Notably, no significant difference in vascular density was observed between the NTDDO group (with inferior alveolar blood vessel ligation) and the CDO group, suggesting that vascular ligation in the NTDDO model did not impair neovascularization within the distraction zone. This phenomenon may be attributed to the abundant collateral blood supply in craniofacial bones. Residual vascular networks from bone stumps and the highly vascularized periosteum likely adequately perfuse the distraction gap.

 Mechanistically, studies indicate that sprouting angiogenesis, characterized by vascular outgrowth from pre-existing vessels, represents the predominant mode of vasculogenesis coupled with osteogenesis [23]. Vascular columns originating from the periosteum and bone stumps were observed to align radially toward the central regenerate [24]. The periosteum, endowed with a dense capillary system, serves as a critical reservoir for bone-associated vascularization [25].

 Runx2 is essential for chondrocyte maturation and osteoblast differentiation [26]. The early differentiation of osteoblasts is primarily governed by Runx2. Genetic ablation of Runx2 in osteoblasts completely suppresses differentiation, abolishing both periosteal and endochondral ossification [27]. During the initial phase of osteoblast differentiation, Runx2 activates the expression of bone matrix proteins, including type I collagen, osteopontin, osteocalcin, and bone sialoprotein [28]. Runx2 expression peaks in pre-osteoblasts/immature osteoblasts and declines in mature osteoblasts, underscoring its critical role in early differentiation [29].

In this study, Runx2 staining of NTDDO and CDO specimens at various stages revealed that Runx2-positive cells were diffusely distributed across the fibrous tissue in the central distraction gap at the conclusion of the distraction phase. With prolonged fixation, trabecular bone formation progressed, and the strongest Runx2 expression was observed in osteoblasts at the advancing edges of trabeculae. Notably, young osteocytes embedded within osteoid or adjacent to bone surfaces also exhibited positive staining. These findings align with the immunolocalization patterns of Runx2 protein reported by Amir et al. [30] in human mandibular vertical distraction osteogenesis. Both groups demonstrated robust Runx2 staining without significant differences in intensity or depth, indicating comparable osteogenic activity in NTDDO and CDO.

We established NTDDO and CDO animal models to investigate osteogenesis within the distraction zone. Comprehensive evaluations, including gross specimen observation, radiographic imaging, micro-CT analysis, biomechanical testing, and histological assessment, revealed no significant differences between the two groups in callus formation, bone mineral density (BMD), mechanical properties, or microscopic morphology. These findings collectively indicate that NTDDO achieves osteogenic quality comparable to CDO, with inferior alveolar neurovascular bundle resection exerting no detrimental effects on bone regeneration. This aligns with the findings of Hasse et al. [31], who demonstrated robust callus formation in adult animal models following complete osteotomy and bilateral transection of the inferior alveolar neurovascular bundles, with equivalent new bone formation observed at both proximal and distal edges of the distraction gap.

 However, conflicting evidence exists regarding the impact of neurovascular disruption on osteogenesis. Cao et al. [14]reported reduced BMD, neovascular volume, and trabecular bone formation in denervated rabbit mandibles following 6 mm inferior alveolar nerve resection (with preserved vasculature), compared to the contralateral control side. Similarly, Tevlin et al. [15]observed diminished histo-radiographic osteogenesis and impaired skeletal stem cell (SSC) expansion in murine mandibular DO models after 4 mm inferior alveolar nerve resection. These studies attributed osteogenic impairment to permanent sensory nerve ablation, contrasting with our findings. Notably, Isomura et al. [32, 33]demonstrated successful inferior alveolar nerve regeneration in canine bifocal transport DO models, with histologically continuous nerve fibers and restored electrophysiological functionality at 6 months postoperatively. Shogen et al. [34]further confirmed functional nerve recovery via retrograde transport of horseradish peroxidase-labeled neurons in the trigeminal ganglion. In our NTDDO model, micro-CT revealed continuity of the inferior alveolar nerve canal within the distraction gap. However, whether true nerve regeneration or functional recovery occurs in NTDDO, and whether it contributes to the regulation of osteogenesis, remain unclear. Future studies employing dedicated neural assessment tools are needed to investigate these questions.

Furthermore, the periosteum plays a pivotal role in bone repair and regeneration by supplying oxygen, minerals, and osteoprogenitor cells capable of differentiating into osteoblasts under mechanical stimulation [35, 36]. Unlike CDO, where periosteal regeneration occurs gradually during distraction, the NTDDO model preserves intact periosteal coverage around bone stumps post-segmental mandibulectomy. This retained periosteal reservoir likely enhances osteogenic efficiency, potentially compensating for neurovascular alterations.

The feasibility of NTDDO was initially demonstrated in a clinical case [7]. The present animal study provides experimental evidence of its osteogenic equivalence to CDO, supporting further clinical evaluation in strictly selected patients with small, benign mandibular defects located in the mandibular body or symphysis. Before widespread clinical application, larger-scale clinical studies with longer follow-up are still needed to confirm its safety and efficacy.

## Conclusions

 Non-transport disc distraction osteogenesis achieves osteogenic quality equivalent to conventional distraction osteogenesis, with inferior alveolar neurovascular resection posing no adverse effects on bone regeneration. The underlying mechanisms, particularly the potential interplay between nerve regeneration and periosteal contributions, warrant further exploration.

## Data Availability

The datasets used and/or analyzed during the current study are available from the corresponding author upon reasonable request.
